# Pulmonary embolism severity before and during the COVID-19 pandemic

**DOI:** 10.1259/bjr.20210264

**Published:** 2021-06-09

**Authors:** Vicky Tilliridou, Rachael Kirkbride, Rebecca Dickinson, James Tiernan, Guo Liang Yong, Edwin JR van Beek, John T Murchison, Michelle Claire Williams

**Affiliations:** 1 Department of Radiology, Royal Infirmary of Edinburgh, Edinburgh, UK; 2 Department of Respiratory Medicine, Royal Infirmary of Edinburgh, Edinburgh, UK; 3 Edinburgh Imaging, University of Edinburgh, Edinburgh, UK; 4 BHF/University of Edinburgh Centre for Cardiovascular Science, Edinburgh, UK

## Abstract

**Objectives:**

Early in the coronavirus 2019 (COVID-19) pandemic, a high frequency of pulmonary embolism was identified. This audit aims to assess the frequency and severity of pulmonary embolism in 2020 compared to 2019.

**Methods:**

In this retrospective audit, we compared computed tomography pulmonary angiography (CTPA) frequency and pulmonary embolism severity in April and May 2020, compared to 2019. Pulmonary embolism severity was assessed with the Modified Miller score and the presence of right heart strain was assessed. Demographic information and 30-day mortality was identified from electronic health records.

**Results:**

In April 2020, there was a 17% reduction in the number of CTPA performed and an increase in the proportion identifying pulmonary embolism (26%, *n* = 68/265 *vs* 15%, *n* = 47/320, *p* < 0.001), compared to April 2019. Patients with pulmonary embolism in 2020 had more comorbidities (*p* = 0.026), but similar age and sex compared to 2019. There was no difference in pulmonary embolism severity in 2020 compared to 2019, but there was an increased frequency of right heart strain in May 2020 (29 *vs* 12%, *p* = 0.029). Amongst 18 patients with COVID-19 and pulmonary embolism, there was a larger proportion of males and an increased 30 day mortality (28% *vs* 6%, *p* = 0.008).

**Conclusion:**

During the COVID-19 pandemic, there was a reduction in the number of CTPA scans performed and an increase in the frequency of CTPA scans positive for pulmonary embolism. Patients with both COVID-19 and pulmonary embolism had an increased risk of 30-day mortality compared to those without COVID-19.

**Advances in knowledge:**

During the COVID-19 pandemic, the number of CTPA performed decreased and the proportion of positive CTPA increased. Patients with both pulmonary embolism and COVID-19 had worse outcomes compared to those with pulmonary embolism alone.

## Introduction

Since its initial emergence in December 2019 in Wuhan city (Hubei Province, China) the SARS-coronavirus-2 (SARS-CoV-2) has spread around the world causing a febrile respiratory illness. However, early in the coronavirus 2019 (COVID-19) disease pandemic reports emerged regarding a high frequency of pulmonary embolism in hospitalised patients with COVID-19.^1–3^ The frequency of pulmonary embolism in patients with COVID-19 of varying severities remains uncertain. However, it is of note that this all occurred at the same time as hospital attendances for non-COVID-19 conditions were also reduced.^4^

The pathophysiology driving the increased rate of thromboembolism in COVID-19 patients is incompletely understood. Risk factors for pulmonary embolism in COVID-19 patients include elevated d-dimer, absence of anticoagulation, intensive care admission, male gender, increased C-reactive protein and increased time from symptom onset to hospitalisation.^5,6^ However, it has been postulated to be secondary to endotheliitis and immune system activation leading to clotting cascade activation and immunothrombosis.^7–9^ In addition to macrothrombosis, post-mortem studies have found microvascular thrombi in the lungs and other organs throughout the body in patients with COVID-19.^10,11^ An alternative proposed mechanism for pulmonary embolism in COVID-19 patients is ‘*in situ*’ thrombosis secondary to inflammation, rather than conventional thromboembolic patterns of disease.^12^ There is emerging evidence describing increased rates of smaller peripheral thrombi and pulmonary thrombosis without associated deep vein thrombosis in patients with COVID-19,^13^ with the suggestion of COVID-19 patients having their own ‘unique pulmonary embolism phenotype’.^14^

In light of these findings, this audit aimed to assess whether there was an increase in the frequency and severity of pulmonary embolism in patients being investigated for suspected pulmonary embolism in 2020, with and without COVID-19, compared to 2019.

## Methods

### Study design

Over a period of 8 weeks during the first peak of the COVID-19 pandemic (1 April 2020–30 May 2020), we performed a retrospective audit of all patients undergoing computed tomography pulmonary angiography (CTPA) at three hospitals providing radiological services for a population of 897,770 (Royal Infirmary of Edinburgh, Western General Hospital Edinburgh, St John’s Hospital Livinigston). All three hospitals were general teaching hospitals situated with in urban settings, with two city-based hospitals and one in a large adjacent town. We also collected data from the same 8-week period in 2019. In this area, the first “lock down” began on 24 March 2020 and easing of restrictions began on 29 May 2020. Ethical approval and informed consent were not required for this retrospective audit. Local audit approval was obtained.

### CTPA

**Figure 1.**
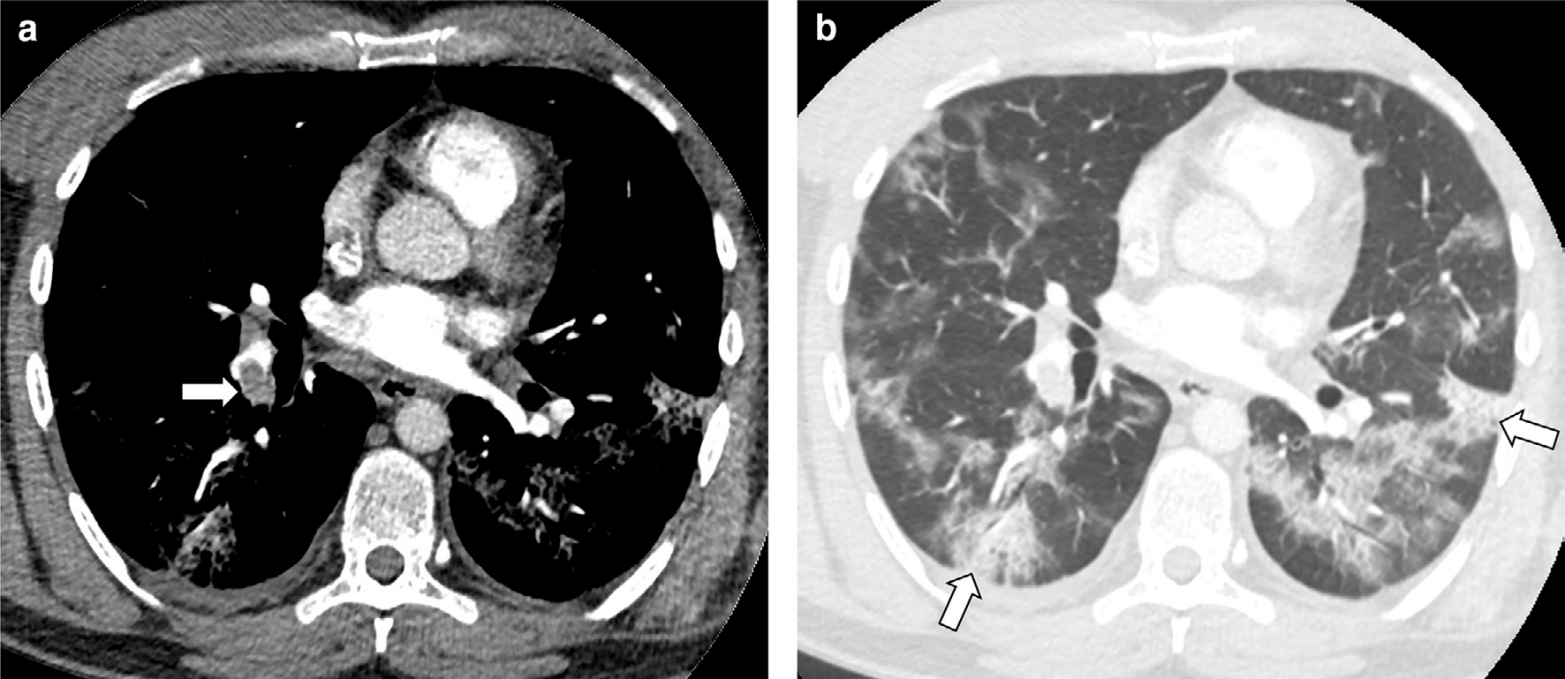
Example images from a patient with COVID-19 showing (**A**) pulmonary embolism on vascular windowed images and (**B**) peripheral ground glass opacification and consolidation on lung windowing in a typical pattern.

The radiological reports for all CTPA performed at the three hospitals were reviewed. Assessment of imaging was performed blinded to COVID-19 status. For patients with acute pulmonary embolism reported on CTPA, the radiological images were reviewed by trained observers (by three radiology trainees with between 4 and 5 years experience and two thoracic subspecialist consultant radiologists with >10 and >20 years experience, with challenging cases reviewed by consensus). Pulmonary embolism severity was calculated using the Modified Miller score. The ratio of the right ventricle to left ventricle diameter (RV/LV ratio) was calculated to assess right heart strain. An RV/LV ratio above 1.2 was considered indicative of right heart strain.^15^ CT lung parenchymal appearances were classified according to the Radiological Society of North America (RSNA) expert consensus statement^16^ as Typical, Indeterminate, Atypical and Negative (Figure 1).

## Clinical information

Information was obtained from electronic medical records regarding age, sex, referring location, past medical history and COVID-19 reverse transcription polymerase chain reaction (RT-PCR) test result. The Charlson comorbidity index was used to assess the presence of comorbidities.^17^ Mortality at 30 days was recorded from the electronic medical records.

## Statistical analysis

Statistical analysis was performed using R, v. 3.5.0 (R Foundation for Statistical Computing, Vienna, Austria). Normally distributed variables are presented as mean ± standard deviation. Non-normally distributed data are presented as median and interquartile range [IQR]. Statistical significance was derived using Pearson χ^2^ test, Wilcoxon test or Student’s *t*-test, as appropriate. A two-tailed *p*-value < 0.05 was considered statistically significant.

## Results

### CTPA frequency

There was a 17% reduction in the number of CTPA performed in April 2020 (265 *vs* 320) and an 0.8% increase in May 2020 (353 *vs* 350), compared to the same months in 2019. There were some differences between the number of CTPA performed in the three hospitals, with the reduction in April 2020 ranging from 16 to 23% and the change in May 2020 varying from an increase of 7% to a decrease of 8% (Supplementary Material 1). In April 2020, there was an increase in the proportion of CTPA requests from intensive care wards and the emergency department (6 *vs* 3% and 22 vs 19%, respectively) and a decrease from inpatient wards and outpatient departments (69 *vs* 73% and 2 vs 6%, *p* = 0.004), compared to April 2019.

Supplementary Material 1.

### CTPA with pulmonary embolism

For the 242 patients with pulmonary embolism on CTPA, the mean age was 62 ± 17 years, 60% were male and the median Charlson comorbidity score was 3 [IQR 1–5]. There was no difference in age or gender in patients with pulmonary embolism on CTPA in April or May 2020 compared to 2019 (Table 1). However, patients with pulmonary embolism had fewer comorbidities in April 2020 compared to April 2019 (Charlson comorbidity score 2 [IQR IQR 1–4] *vs* 4 [IQR 1–6], *p* = 0.026).

**Table 1.** Demographic information, CT findings and outcomes for patients with pulmonary embolism in April/May 2019 and 2020

		April **2019**	April 2020	*p*	May 2019	May **2020**	*p*
n		47	68	-	68	59	-
Age		65 *±* 14	60 *±* 16	0.058	63 *±* 17	61 *±* 18	0.608
Male (%)		27 (57)	46 (68)	0.358	34 (50)	37 (63)	0.208
Charlson Comorbidity score		4 [1, 6]	2 [1, 4]	**0.026**	3 [1, 5]	3 [1, 6]	0.605
Modified Miller score		5 [2, 14]	8 [2, 12]^12^	0.553	6 [3, 13]	6 [2, 12]	0.796
RV/LV ratio		0.94 [0.84, 1.17]	0.93 [0.85, 1.07]	0.718	0.92 [0.85, 1.05]	0.97 [0.83, 1.21]	0.306
Right heart strain *		10 (21)	9 (13)	0.376	8 (12)	17 (29)	**0.029**
CT COVID-19	Typical	2 (4)	27 (40)	**<0.001**	4 (6)	11 (19)	**0.047**
	Indeterminate	2 (4)	6 (9)		14 (21)	7 (12)	
	Atypical	14 (30)	12 (18)		22 (32)	12 (20)	
	Negative	29 (62)	23 (34)		28 (41)	29 (49)	

LV, left ventricle; RV, right ventricle.

Number (%); Mean ± standard deviation; Median [Interquartile range].

a RV/LV ratio >1.2.

**Figure 2.**
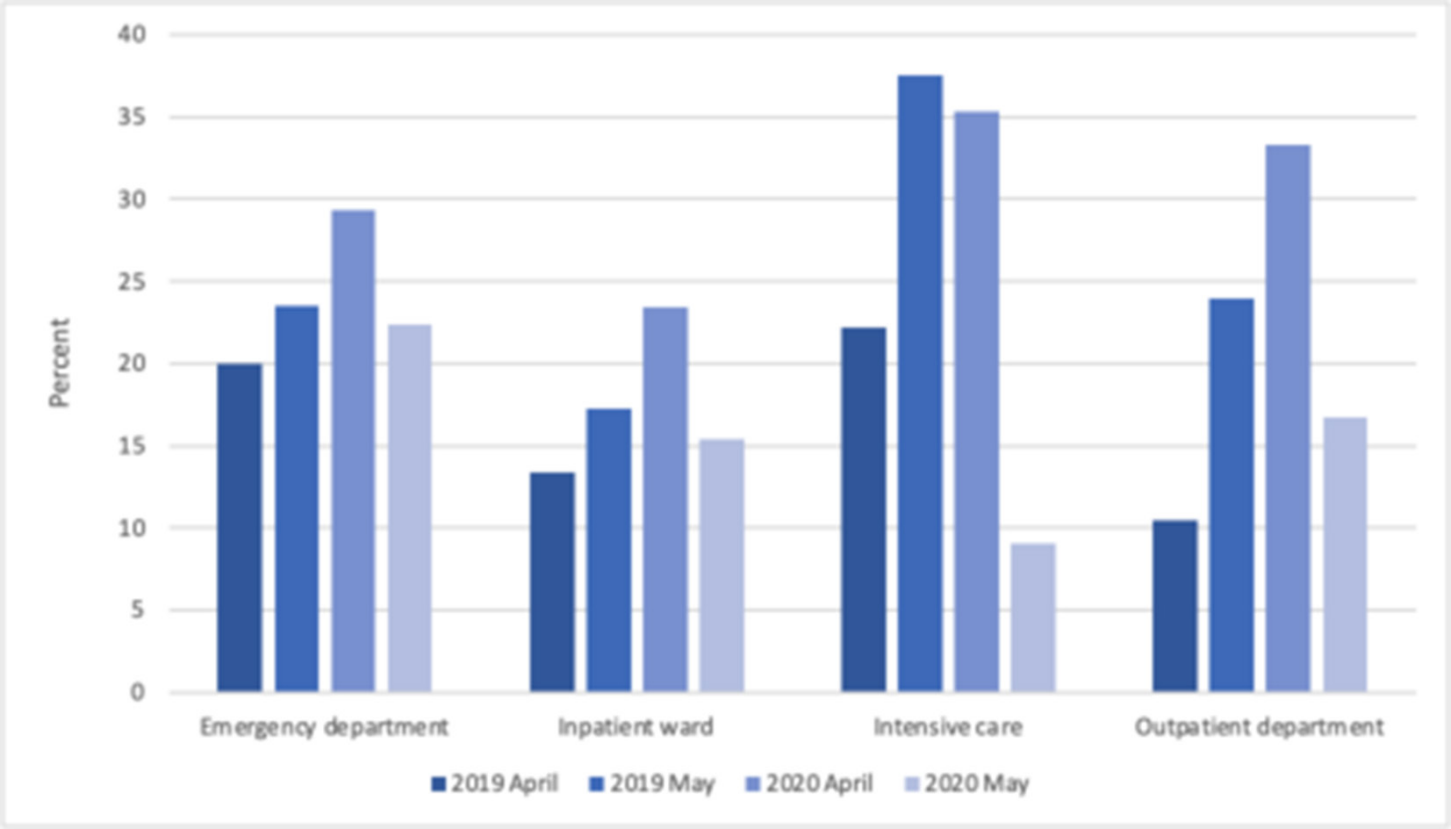
Percentage of CTPA positive for pulmonary embolism by requesting location, comparing April and May 2019 to 2020. CTPA, computed tomography pulmonary angiography.

There was an increase in the proportion of CTPA with pulmonary embolism in April 2020 compared to April 2019 (26%, *n* = 68/265 *vs* 15%, *n* = 47/320, *p* < 0.001), but there was no difference in May 2020 compared to May 2019 (17%, *n* = 59/353 *vs* 19%, *n* = 68/350, *p* = 0.402). The proportion of positive CTPA requested from inpatient wards was higher in April 2020 compared to April 2019 (23 *vs* 13%, *p* = 0.047, Figure 2), but the increase in emergency department (29% *vs* 20% *p* = 0.673) and outpatient department requests (33% *vs* 11%, *p* = 0.552) was not statistically significant. There was no statistically significant difference in the proportion of positive CTPA across the three hospitals (Supplementary Material 1).

**Figure 3.**
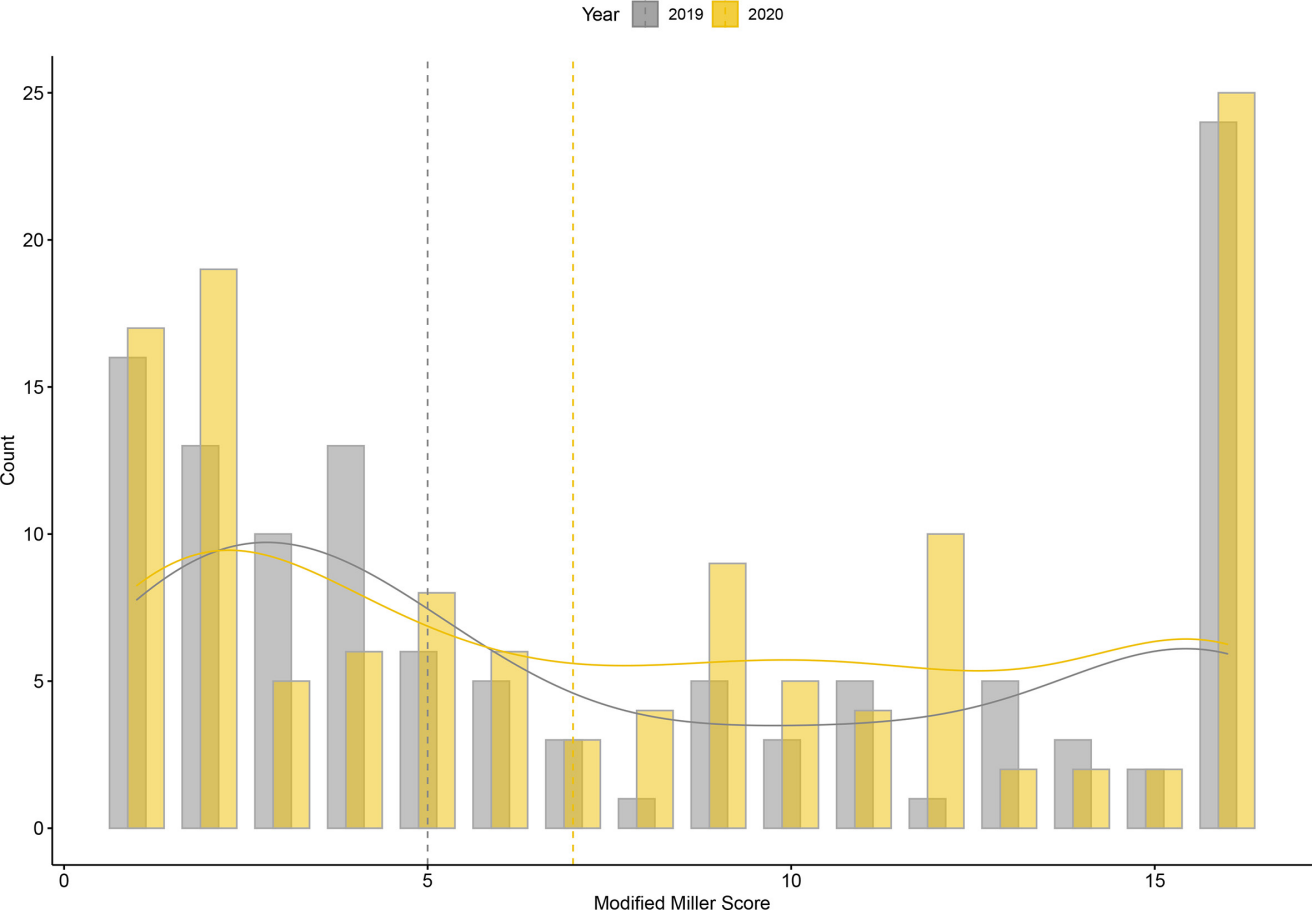
Pulmonary embolism severity assessed with the Modified Miller Score in 2019 (grey) and 2020 (yellow). The smoothed density estimates are shown by the solid lines and the medians by the vertical dotted lines.

There was no difference in pulmonary embolism severity between 2019 and 2020 in terms of Modified Miller score or RV/LV ratio (Figure 3). However, patients presenting in May 2020 had an increased frequency of right heart strain evidenced by an RV/LV ratio above the ‘tipping point’ of 1.2 (29% *vs* 12%, *p* = 0.029, Table 1). In 2019, patients with pulmonary embolism in one hospital (with a higher proportion of cancer and medicine of the elderly inpatients) had more comorbidities and more severe pulmonary embolism (Supplementary Material 1). However, this difference was not apparent in 2020. Information on anticoagulation was available for a subset of patients with pulmonary embolism from one hospital. Prophylactic anticoagulation was administered to 50% of patients with pulmonary embolism in 2019 (*n* = 4/8), 100% of patients with non-COVID-19 related pulmonary embolism in 2020 (*n* = 3/3) and 86% of COVID-19-related pulmonary embolism in 2020 (*n* = 6/7). Prophylactic anticoagulation was not administered to one patient with COVID-19 who developed pulmonary embolism due to a significant underlying bleeding risk.

A “Typical” COVID-19 appearance was apparent on a higher frequency of CTPA in April and May 2020 compared to 2019 (40%, *n* = 27 *vs* 4%, *n* = 2, *p* < 0.001 and 19%, *n* = 11 *vs* 4%, *n* = 4, *p* = 0.052, respectively; Table 1). However, in April and May 2019, 5% of patients had a “Typical” appearance, 14% had an “Indeterminate” appearance and 31% had an “Atypical” appearance, despite these studies predating the COVID-19 pandemic.

## COVID-19 patients

Of the 127 patients with a positive CTPA in 2020, 18 (14%) had a diagnosis of COVID-19 by RT-PCR. Patients with pulmonary embolism and a diagnosis of COVID-19 in 2020 had a similar age and Charlson comorbidity score compared to patients with pulmonary embolism in 2019 (Table 2). However, there was a larger proportion of males amongst patients with pulmonary embolism and COVID-19 compared to those with pulmonary embolism in 2019 (83% *vs* 53%, *p* = 0.031). There was no difference in pulmonary embolism severity or the presence of right heart strain between these groups. Patients with pulmonary embolism and COVID-19 were more likely to have a “Typical” CT appearance (67% *vs* 5%, *p* < 0.001) compared to those with pulmonary embolism in 2019.

**Table 2.** Demographic information, CT findings and outcomes for patients with pulmonary embolism and COVID-19 in 2020 compared to those with pulmonary embolism in 2019

		Pulmonary embolism in 2019	Pulmonary embolism and COVID-19 in 2020	P
Number		115	18	
Age (years)		64 *±* 16	64 *±* 16	0.923
Male (%)		61 (53)	15 (83)	**0.031**
Charlson comorbidity score		3 [1, 5]	3 [1, 4]	0.713
Modified Miller score		5 [2, 14]	5 [2, 11]	0.497
RV/LV ratio		0.93 [0.85 to 1.07]	0.94 [0.86 to 1.03]	0.755
Right heart strain *		18 (16)	4 (22)	0.721
CT COVID-19	Typical	6 (5)	12 (67)	**<0.001**
	Atypical	36 (31)	2 (11)	
	Indeterminate	16 (14)	1 (6)	
	Negative	57 (50)	3 (17)	

LV, left ventricle; RV, right ventricle.

Number (%); Mean ± standard deviation; Median [Interquartile range].

a RV/LV ratio >1.2

## Mortality

**Figure 4.**
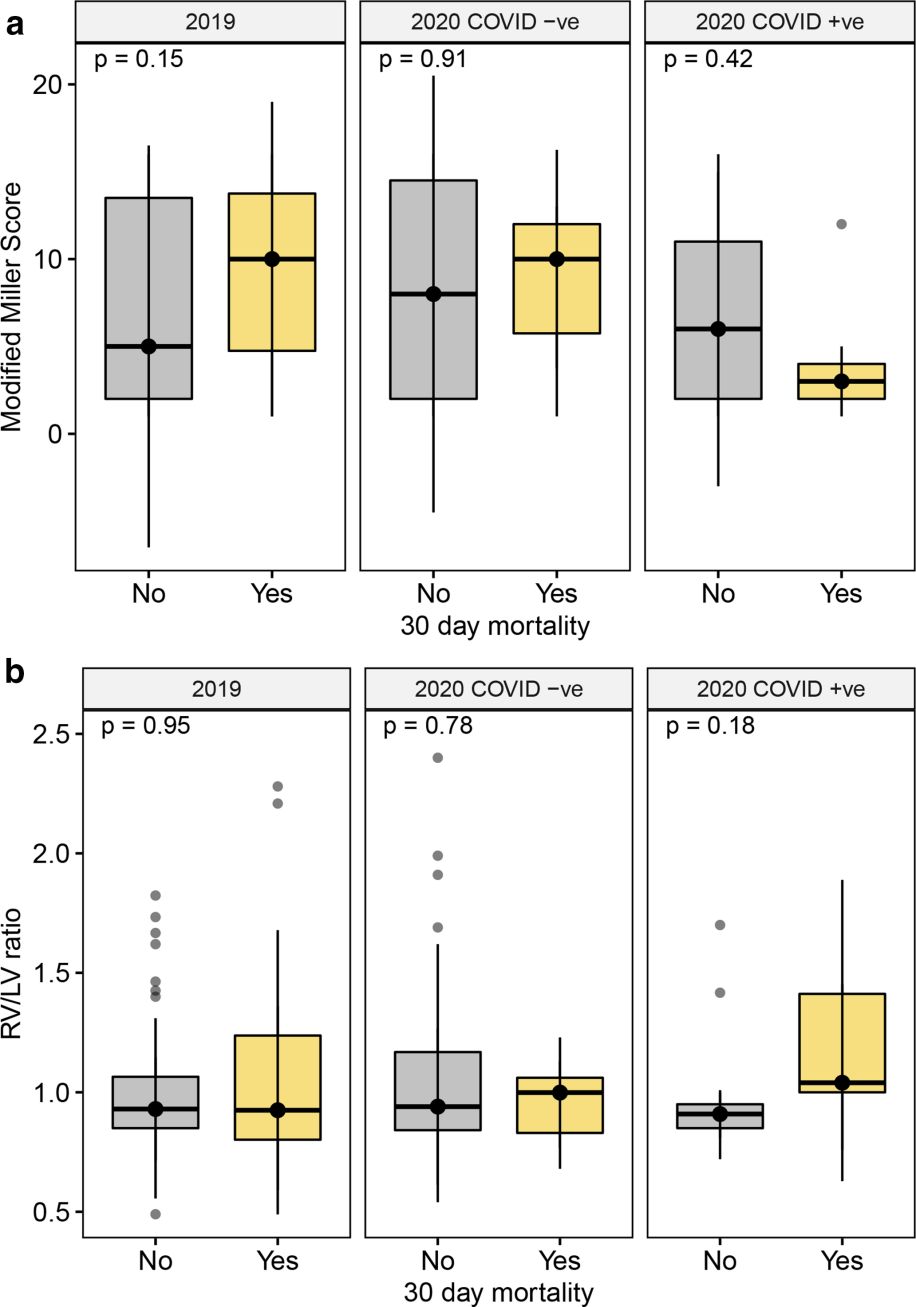
Modified Miller score (**A**) and RV/LV ratio (**B**) in patients with and without mortality at 30 days, comparing patients with pulmonary embolism in 2019 and pulmonary embolism with and without COVID-19 in 2020 (Median and interquartile range). LV, left ventricle; RV, right ventricle.

For all patients with pulmonary embolism, the 30-day mortality was similar in 2020 compared to 2019 (9% *vs* 14%, *p* = 0.275). However, in 2020 patients with both pulmonary embolism and a diagnosis of COVID-19 had worse outcomes, with mortality at 30 days occurring in 28% compared to 6% in those without COVID-19 (*n* = 5/18 *vs n* = 6/109, *p* = 0.008). Patients with COVID-19 who died within 30 days had a lower Modified Miller score and higher RV/LV ratio compared to those without COVID-19, although these differences were not statistically significant (Figure 4).

## Discussion

We have shown that during the first wave of the COVID-19 pandemic in 2020, there was a reduction in the number of CTPA scans performed and an increase in the proportion that were positive for pulmonary embolism. Patients presenting with pulmonary embolism in April 2020 had fewer comorbidities compared to the previous year but had similar pulmonary embolism severity. Patients with both pulmonary embolism and COVID-19 were more likely to be male and had an increased 30-day mortality. These findings highlight the different characteristics of patients presenting with COVID-19 and pulmonary embolism, as well as the impact of the COVID-19 pandemic on the diagnosis of pulmonary embolism.

The reduction in the number of CTPA performed during the first peak of the pandemic has important implications for patients. Studies of other diagnostic tests have similarly found a reduction during the COVID-19 pandemic, including an 88% reduction in endoscopy,^18^ a 60% reduction in breast cancer screening^19^ and a worldwide 64% reduction in cardiac imaging.^20^ Between March and August 2020, in England there was a 35% reduction in key diagnostic tests compared to 2019.^21^ Identifying the correct diagnosis is central to clinical care, and the reduction in diagnostic tests may lead to incorrect or delayed diagnoses. We also noted a change in referral patterns, with an increase in the number of requests from intensive care wards and emergency departments, and a decrease in requests from inpatient wards and outpatient departments, likely representing changes in the delivery of health care during the pandemic. There are a variety of potential reasons for the reduction in the number of CTPA performed during the COVID-19 pandemic. This includes patients not attending hospital due to fear or infection, lack of transportation or other concerns. In addition, “breathlessness” may have been incorrectly attributed to COVID-19 rather than pulmonary embolism. There is also an unknown number of patients who may have died with pulmonary embolism without attending hospital or undergoing imagining, both with and without COVID-19. For future waves of the pandemic, it is important that we develop strategies that will allow us to maintain good clinical care for patients with non-COVID-19 diagnoses, and facilitating diagnostic testing is essential for this.

Similar to previous studies, we found an increased positivity rate for CTPA during the first wave of the pandemic in April 2020. One early study from New York found that 37.1% of CTPA in COVID-19 patients showed pulmonary embolism, increased from a rate of 14.5% pre-COVID-19.^22^ Amongst non-critical care COVID-19 patients in Madrid, Spain in March/April 2020, Mestre-Gomez et al found a 6.4% incidence of pulmonary embolism, despite appropriate use of prophylactic anticoagulation.^23^ In one hospital in France in March/April 2020, pulmonary embolism was identified in 23% of patients with COVID-19, with associations with mechanical ventilation and male sex on multivariable analysis.^24^ However, the true frequency of pulmonary embolism in patients with COVID-19 remains unknown. Cohort studies of hospitalised patients have identified pulmonary embolism in between 2 and 57% of patients.^2,6,13,25–28^ A meta-analysis of studies published between January and June 2020 found that pulmonary embolism occurred in 16.5% of patients with COVID-19, with an increased frequency in patients admitted to intensive care units.^13^ However, a recent multicentre retrospective study of 39,408 patients attending Spanish and French emergency departments during the COVID-19 pandemic, and 97,194 patients attending during the pre-COVID-19 era, found that once a diagnosis of pulmonary embolism was suspected, pulmonary embolism occurred in a similar frequency in COVID-19 and non-COVID-19 patients.^29^ They identified pulmonary embolism in 0.7% of patients with COVID-19 and the risk of emergency department patients having pulmonary embolism was seven times higher in the COVID-19 era compared to the pre-COVID-19 era.^29^ The prevalence of out of hospital pulmonary embolism and subclinical disease is unknown, and other studies have shown that non-hospitalised patients with COVID-19 are also at risk of pulmonary embolism.^30,31^ The proportion of CTPA with pulmonary embolism in our study returned to almost normal levels in May 2020, which may represent the return to normal imaging frequencies, improved anticoagulation, reduction in the number of COVID-19 cases, or lifestyle changes associated with the end of restrictions to movement. Tsakok et al found that in one UK hospital, there was a threefold increase in the number of CTPA performed during the second wave of the pandemic, possibly highlighting an increased awareness of potential thromboembolic complications of COVID-19.^32^

With the potential thrombogenic effects of COVID-19, we hypothesised that there would be an increase in the proportion of positive CTPA in 2020, but this may also be secondary to the decrease in the number of CTPA performed, with only more unwell patents undergoing imaging. However, we found a similar range of severity of pulmonary embolism between 2020 and 2019 in terms of the Modified Miller Scores. Therefore, it is possible that there was an increase in the number of low severity pulmonary embolism during the COVID-19 pandemic, due to the thrombogenic effects of COVID-19, the effects of immobility, or other unknown factors. This is similar to the findings of van Dam et al who showed that 23 patients with pulmonary embolism and COVID-19 had lower thrombus load and less right heart strain compared to 100 pre-pandemic controls with pulmonary embolism.^12^ However, in May 2020 we identified an increased frequency of right heart strain on CTPA compared to 2019, potentially suggesting the presentation of more unwell patients as the pandemic evolved.

Although we found no difference in pulmonary embolism severity in patients with COVID-19, this does not exclude an the presence of microthrombi in COVID-19 patients. Several histopathology studies describe microthrombi within small pulmonary vessels in patients with COVID-19,^10,11^ with a systemic review showing that microthrombi were found in 54% of COVID-19 patients compared to 24% of H1N1 influenza patients.^33^ Autopsy findings from patients with COVID-19 have shown diffuse alveolar damage in the lungs, along with macro- and microscopic thrombosis affecting in particular small to mid-sized pulmonary arteries.^34,35^ Interestingly, traditional thromboembolic risk factors are not associated with the occurrence of pulmonary embolism in patients with COVID-19.^6^ Pulmonary vascular abnormalities, such as dilated distal pulmonary arteries, have been identified in patients with COVID-19, which may be associated with pulmonary microthrombi.^36^ Such small pulmonary emboli may not be visualised on CTPA, particularly when associated with suboptimal image quality and lung parenchymal features of COVID-19. A study of 4389 hospitalised patients with COVID-19 found that those receiving anticoagulation had a lower mortality and reduced ventilator requirements compared to those not receiving anticoagulation, but with no differences between those receiving therapeutic and prophylactic doses.^37^ A recent study demonstrated that patients receiving anticoagulant therapy prior to admission to hospital for COVID-19 had better outcomes.^38^ Routine prophylactic anticoagulation is now part of clinical care for hospitalised patients with COVID-19,^39^ however, the nature of the link between COVID-19 and thromboembolism remains uncertain.^40^

The RSNA reporting language for CT findings related to COVID-19 was established to standardise reporting and aid communication of findings. They have previously been shown to have a sensitivity of 97.5% and specificity of 54.7% for the diagnosis of COVID-19 when typical and indeterminate findings were combined.^41^ However, there are overlaps between the appearance of pulmonary infarction and COVID-19. We have shown up to 20% of patients with pulmonary embolism can have typical or indeterminate CT findings, in the absence of COVID-19. Therefore, it is important to maintain a high clinical suspicion for both pulmonary embolism and COVID-19 when typical or indeterminate findings area identified on CT.

This audit has some limitations. Despite the inclusion of three hospitals, the number of patients testing positive for COVID-19 with pulmonary embolism on CTPA was low. We do not have data on the management of pulmonary embolism and information on prophylactic anticoagulation is only available for a subset of patients in one hospital. Nevertheless, in this small subset of patients, appropriate use of prophylactic anticoagulation was demonstrated. In all three hospitals, standard thromboprophylaxis was provided as per hospital guidelines in 2019 and 2020, with enhanced venous thromboembolism prophylaxis considered for patients in intensive care as the first wave of the pandemic evolved. Patients who died without attending hospital or without undergoing CTPA are not included in this analysis. There may be other reasons why patients did not undergo CTPA, such as patient reluctance to attend hospital during the pandemic. We hypothesise that the larger reduction in CTPA in April 2020 compared to May 2020 was in response to changes in the phase of the pandemic, health-care activities, and public engagement with health-care services. The first “lock down” began in this area on 23 March 2020, with associated dramatic changes in activity in April 2020, followed by gradual return to normal levels across May 2020 and beyond. However, we cannot exclude other difference between these months which may impact the results.

In conclusion, during the COVID-19 pandemic there was a reduction in the number of CTPA scans performed and an increase in the frequency of CTPA scans positive for pulmonary embolism. Patients with both COVID-19 and pulmonary embolism had an increased risk of 30-day mortality compared to those without COVID-19.

## References

[1] Klok FA, Kruip MJHA, van der Meer NJM, Arbous MS, Gommers D, Kant KM, et al. Confirmation of the high cumulative incidence of thrombotic complications in critically ill ICU patients with COVID-19: an updated analysis. Thromb Res 2020; 191: 148–50. doi: 10.1016/j.thromres.2020.04.041 32381264 PMC7192101

[2] Helms J, Tacquard C, Severac F, Leonard-Lorant I, Ohana M, Delabranche X, et al. High risk of thrombosis in patients with severe SARS-CoV-2 infection: a multicenter prospective cohort study. Intensive Care Med 2020; 46: 1089–98. doi: 10.1007/s00134-020-06062-x 32367170 PMC7197634

[3] Poissy J, Goutay J, Caplan M, Parmentier E, Duburcq T, Lassalle F, et al. Pulmonary embolism in patients with COVID-19: awareness of an increased prevalence. Circulation 2020; 142: 184–6. doi: 10.1161/CIRCULATIONAHA.120.047430 32330083

[4] Wyatt S, Mohammed MA, Fisher E, McConkey R, Spilsbury P. Impact of the SARS-CoV-2 pandemic and associated lockdown measures on attendances at emergency departments in English hospitals: a retrospective database study. The Lancet Regional Health - Europe. 10.1016/j.lanepe.2021.100034 PMC7837109 34173630

[5] Mouhat B, Besutti M, Bouiller K, Grillet F, Monnin C, Ecarnot F, et al. Elevated D-dimers and lack of anticoagulation predict PE in severe COVID-19 patients. Eur Respir J 2020; 56: 2001811 22 10 2020. doi: 10.1183/13993003.01811-2020 32907890 PMC7487272

[6] Fauvel C, Weizman O, Trimaille A, Mika D, Pommier T, Pace N, et al. Pulmonary embolism in COVID-19 patients: a French multicentre cohort study. Eur Heart J 2020; 41: 3058–68. doi: 10.1093/eurheartj/ehaa500 32656565 PMC7528952

[7] Jayarangaiah A, Kariyanna PT, Chen X, Jayarangaiah A, Kumar A. COVID-19-Associated coagulopathy: an exacerbated Immunothrombosis response. Clin Appl Thromb Hemost 2020; 26: 107602962094329. doi: 10.1177/1076029620943293 32735131 PMC7401047

[8] Teuwen L-A, Geldhof V, Pasut A, Carmeliet P. COVID-19: the vasculature unleashed. Nat Rev Immunol 2020; 20: 389–91. doi: 10.1038/s41577-020-0343-0 32439870 PMC7240244

[9] Ackermann M, Verleden SE, Kuehnel M, Haverich A, Welte T, Laenger F, et al. Pulmonary vascular Endothelialitis, thrombosis, and angiogenesis in Covid-19. N Engl J Med 2020; 383: 120–8. doi: 10.1056/NEJMoa2015432 32437596 PMC7412750

[10] Lodigiani C, Iapichino G, Carenzo L, Cecconi M, Ferrazzi P, Sebastian T, et al. Venous and arterial thromboembolic complications in COVID-19 patients admitted to an academic hospital in Milan, Italy. Thromb Res 2020; 191: 9–14. doi: 10.1016/j.thromres.2020.04.024 32353746 PMC7177070

[11] Geng Y-J, Wei Z-Y, Qian H-Y, Huang J, Lodato R, Castriotta RJ. Pathophysiological characteristics and therapeutic approaches for pulmonary injury and cardiovascular complications of coronavirus disease 2019. Cardiovasc Pathol 2020; 47: 107228. doi: 10.1016/j.carpath.2020.107228 32375085 PMC7162778

[12] van Dam LF, Kroft LJM, van der Wal LI, Cannegieter SC, Eikenboom J, de Jonge E, et al. Clinical and computed tomography characteristics of COVID-19 associated acute pulmonary embolism: a different phenotype of thrombotic disease? Thromb Res 2020; 193: 86–9. doi: 10.1016/j.thromres.2020.06.010 32531548 PMC7274953

[13] Suh YJ, Hong H, Ohana M, Bompard F, Revel M-P, Valle C, et al. Pulmonary embolism and deep vein thrombosis in COVID-19: a systematic review and meta-analysis. Radiology 2021; 298: E70–80. doi: 10.1148/radiol.2020203557 33320063 PMC7745997

[14] Chowdhury JF, Moores LK, Connors JM. Anticoagulation in hospitalized patients with Covid-19. N Engl J Med 2020; 383: 1675–8. doi: 10.1056/NEJMclde2028217 33085867

[15] Wong LF, Akram AR, McGurk S, Van Beek EJR, Reid JH, Murchison JT. Thrombus load and acute right ventricular failure in pulmonary embolism: correlation and demonstration of a "tipping point" on CT pulmonary angiography. Br J Radiol 2012; 85: 1471–6. doi: 10.1259/bjr/22397455 22723513 PMC3500789

[16] Simpson S, Kay FU, Abbara S, Bhalla S, Chung JH, Chung M, et al. Radiological Society of North America expert consensus document on reporting chest CT findings related to COVID-19: endorsed by the Society of thoracic radiology, the American College of radiology, and RSNA. Radiol Cardiothorac Imaging 2020; 2: e200152: e200152. doi: 10.1148/ryct.2020200152 33778571 PMC7233447

[17] Charlson ME, Pompei P, Ales KL, MacKenzie CR. A new method of classifying prognostic comorbidity in longitudinal studies: development and validation. J Chronic Dis 1987; 40: 373–83. doi: 10.1016/0021-9681(87)90171-8 3558716

[18] Rutter MD, Brookes M, Lee TJ, Rogers P, Sharp L. Impact of the COVID-19 pandemic on UK endoscopic activity and cancer detection: a national endoscopy database analysis. Gut 2021; 70: 537–43. doi: 10.1136/gutjnl-2020-322179 32690602

[19] Peng S-M, Yang K-C, Chan WP, Wang Y-W, Lin L-J, Yen AM-F, et al. Impact of the COVID-19 pandemic on a population-based breast cancer screening program. Cancer 2020; 126: 5202–5. doi: 10.1002/cncr.33180 32914864

[20] Einstein AJ, Shaw LJ, Hirschfeld C, Williams MC, Villines TC, Better N, et al. International impact of COVID-19 on the diagnosis of heart disease. J Am Coll Cardiol 2021; 77: 173–85. doi: 10.1016/j.jacc.2020.10.054 33446311 PMC7836433

[21] Greenwood E, Swanton C. Consequences of COVID-19 for cancer care - a CRUK perspective. Nat Rev Clin Oncol 2021; 18: 3–4. doi: 10.1038/s41571-020-00446-0 33097915 PMC7582444

[22] Kaminetzky M, Moore W, Fansiwala K, Babb JS, Kaminetzky D, Horwitz LI, et al. Pulmonary embolism at CT pulmonary angiography in patients with COVID-19. Radiol Cardiothorac Imaging 2020; 2: e200308. doi: 10.1148/ryct.2020200308 33778610 PMC7336753

[23] Mestre-Gómez B, Lorente-Ramos RM, Rogado J, Franco-Moreno A, Obispo B, Salazar-Chiriboga D, et al. Incidence of pulmonary embolism in non-critically ill COVID-19 patients. predicting factors for a challenging diagnosis. J Thromb Thrombolysis 2021; 51: 40–6. doi: 10.1007/s11239-020-02190-9 32613385 PMC7327193

[24] Grillet F, Behr J, Calame P, Aubry S, Delabrousse E. Acute pulmonary embolism associated with COVID-19 pneumonia detected with pulmonary CT angiography. Radiology 2020; 296: E186–8. doi: 10.1148/radiol.2020201544 32324103 PMC7233384

[25] Bilaloglu S, Aphinyanaphongs Y, Jones S, Iturrate E, Hochman J, Berger JS. Thrombosis in hospitalized patients with COVID-19 in a new York City health system. JAMA 2020; 324: 799–801. doi: 10.1001/jama.2020.13372 32702090 PMC7372509

[26] Klok FA, Kruip MJHA, van der Meer NJM, Arbous MS, Gommers DAMPJ, Kant KM, et al. Incidence of thrombotic complications in critically ill ICU patients with COVID-19. Thromb Res 2020; 191: 145–7. doi: 10.1016/j.thromres.2020.04.013 32291094 PMC7146714

[27] Ameri P, Inciardi RM, Di Pasquale M, Agostoni P, Bellasi A, Camporotondo R, et al. Pulmonary embolism in patients with COVID-19: characteristics and outcomes in the Cardio-COVID Italy multicenter study. Clin Res Cardiol 2020;03 Nov 2020. doi: 10.1007/s00392-020-01766-y 33141251 PMC7607374

[28] Léonard-Lorant I, Delabranche X, Séverac F, Helms J, Pauzet C, Collange O, et al. Acute pulmonary embolism in patients with COVID-19 at CT angiography and relationship to D-dimer levels. Radiology 2020; 296: E189–91. doi: 10.1148/radiol.2020201561 32324102 PMC7233397

[29] Miró Òscar, Llorens P, Aguirre A, Lozano L, Beaune S, Roussel M, et al. Association between Covid-19 and pulmonary embolism (AC-19-PE study. Thromb Res 2020; 196: 322–4. doi: 10.1016/j.thromres.2020.09.010 32977131 PMC7481175

[30] Morley NCD, Muir KC, Mirsadraee S, van Beek EJR, Murchison JT. Ten years of imaging for pulmonary embolism: too many scans or the tip of an iceberg? Clin Radiol 2015; 70: 1370–5. doi: 10.1016/j.crad.2015.07.010 26385203

[31] Gervaise A, Bouzad C, Peroux E, Helissey C. Acute pulmonary embolism in non-hospitalized COVID-19 patients referred to CtpA by emergency department. Eur Radiol 2020; 30: 6170–7. doi: 10.1007/s00330-020-06977-5 32518989 PMC7280685

[32] Tsakok MT, Qamhawi Z, Lumley SF, Xie C, Matthews P, Gleeson F, et al. COVID-19 CT pulmonary angiogram examinations and reported pulmonary embolism incidence: comparison between peak first wave and early second wave. Clin Radiol 2021; 76: 310–2. doi: 10.1016/j.crad.2021.02.001 33610286 PMC7862906

[33] Hariri LP, North CM, Shih AR, Israel RA, Maley JH, Villalba JA, et al. Lung histopathology in coronavirus disease 2019 as compared with severe acute respiratory Sydrome and H1N1 influenza: a systematic review. Chest 2021; 159: 73–84. doi: 10.1016/j.chest.2020.09.259 33038391 PMC7538870

[34] Lax SF, Skok K, Zechner P, Kessler HH, Kaufmann N, Koelblinger C, et al. Pulmonary Arterial Thrombosis in COVID-19 With Fatal Outcome : Results From a Prospective, Single-Center, Clinicopathologic Case Series. Ann Intern Med 2020; 173: 350–61. doi: 10.7326/M20-2566 32422076 PMC7249507

[35] Fox SE, Akmatbekov A, Harbert JL, Li G, Quincy Brown J, Vander Heide RS. Pulmonary and cardiac pathology in African American patients with COVID-19: an autopsy series from new Orleans. Lancet Respir Med 2020; 8: 681–6. doi: 10.1016/S2213-2600(20)30243-5 32473124 PMC7255143

[36] Lang M, Som A, Carey D, Reid N, Mendoza DP, Flores EJ. Pulmonary vascular manifestations of COVID-19 pneumonia. Radiology: Cardiothoracic Imaging 2020; 2: e200277. 34036264 10.1148/ryct.2020200277 PMC7307217

[37] Nadkarni GN, Lala A, Bagiella E, Chang HL, Moreno PR, Pujadas E, et al. Anticoagulation, bleeding, mortality, and pathology in hospitalized patients with COVID-19. J Am Coll Cardiol 2020; 76: 1815–26. doi: 10.1016/j.jacc.2020.08.041 32860872 PMC7449655

[38] Chocron R, Galand V, Cellier J, Gendron N, Pommier T, Bory O. Anticoagulation prior to hospitalization is a potential protective factor for COVID-19: insight from a French multicenter cohort study. J Am Heart Assoc 2021;: e018288: e018288. 10.1161/JAHA.120.018624 PMC8174166 33550816

[39] Oudkerk M, Büller HR, Kuijpers D, van Es N, Oudkerk SF, McLoud T, et al. Diagnosis, prevention, and treatment of thromboembolic complications in COVID-19: report of the National Institute for public health of the Netherlands. Radiology 2020; 297: E216–22. doi: 10.1148/radiol.2020201629 32324101 PMC7233406

[40] Hammer MM, Raptis CA. COVID-19 and pulmonary thromboembolism. Radiology 2021; 204709. 10.1148/radiol.2021204709 PMC7944848 33687290

[41] Som A, Lang M, Yeung T, Carey D, Garrana S, Mendoza DP, et al. Implementation of the radiological Society of North America expert consensus guidelines on reporting chest CT findings related to COVID-19: a Multireader performance study. Radiol Cardiothorac Imaging 2020; 2: e200276: e200276. doi: 10.1148/ryct.2020200276 33778625 PMC7484923

