# Supplementary material for: Pulmonary embolism severity before and during the COVID-19 pandemic
Source: Br J Radiol. 2021 Jun 9;94(1123):20210264. doi: 10.1259/bjr.20210264 (PMC8248223; doi:10.1259/bjr.20210264)
Supplement: Supplementary Material 1. [file bjr.20210264.suppl-01.docx]

**Pulmonary embolism frequency, severity and mortality before and during the COVID-19 pandemic**

**Supplementary Information**

*Supplementary Table 1: Number of CTPA performed in each hospital and positivity rate.*

| **Year** | **Parameter** | **Hospital 1** | **Hospital 2** | **Hospital 3** | **p** |
| --- | --- | --- | --- | --- | --- |
| 2019 | Number of CTPA | 322 | 133 | 215 | - |
|  | Positive CTPA | 53 (16.5) | 26 (19.5) | 36 (16.7) | 0.715 |
| April 2020 | Number of CTPA | 131 | 43 | 91 | - |
|  | Percent change * | 16% reduction | 23% reduction | 16% reduction | - |
|  | Positive CTPA | 36 (27.5) | 10 (23.3) | 22 (24.2) | 0.793 |
| May 2020 | Number of CTPA | 178 | 71 | 104 | - |
|  | Percent change * | 7% increase | 8% reduction | 3% reduction | - |
|  | Positive CTPA | 32 (18.0) | 12 (16.9) | 15 (14.4) | 0.742 |

*Number (%)*

** Percent reduction compared to the same month in 2019.*

*Supplementary Table 2: Demographic information, CT findings and outcomes for patients with pulmonary embolism in April/May 2019 and 2020 in the 3 hospitals.*

| **Year** | **Parameter** | | **Hospital 1** | **Hospital 2** | **Hospital 3** | **p** |
| --- | --- | --- | --- | --- | --- | --- |
| 2019 | n | | 53 | 26 | 36 | - |
|  | Age | | 65 *±* 16 | 59 *±* 13 | 66 *±* 16 | 0.151 |
|  | Male (%) | | 30 (57) | 13 (50) | 18 (50) | 0.779 |
|  | Charlson Comorbidity score | | 2 [1, 4] | 2 [1, 5] | 4 [3, 6] | **0.007** |
|  | Modified Miller score | | 4 [2, 9] | 7 [3, 14] | 11 [5, 16] | **0.001** |
|  | RV/LV ratio | | 0.93  [0.84, 1.03] | 0.88  [0.78, 1.13] | 0.98  [0.89, 1.21] | **0.045** |
|  | Right heart strain * | | 3 (6) | 6 (23) | 9 (25) | **0.024** |
|  | CT  COVID-19 | Typical | 4 (8) | 2 (8) | 0 (0) | 0.420 |
|  |  | Indeterminate | 9 (17) | 4 (15) | 3 (8) |  |
|  |  | Atypical | 14 (26) | 10 (38) | 12 (33) |  |
|  |  | Negative | 26 (49) | 10 (38) | 21 (58) |  |
| April 2020 | n | | 36 | 10 | 22 | - |
|  | Age | | 57 *±* 16 | 63 *±* 14 | 62 *±* 17 | 0.373 |
|  | Male (%) | | 27 (75) | 5 (50) | 14 (64) | 0.29 |
|  | Charlson Comorbidity score | | 2 [1, 2] | 4 [1, 5] | 3 [1, 5] | **0.041** |
|  | Modified Miller score | | 9 [4, 12] | 7 [2, 16] | 9 [2, 12] | 0.971 |
|  | RV/LV ratio | | 0.90  [0.85, 1.01] | 0.93 [  0.88, 1.05] | 1.00  [0.87, 1.15] | 0.382 |
|  | Right heart strain * | | 3 (8) | 1 (10) | 5 (23) | 0.277 |
|  | CT  COVID-19 | Typical | 5 (14) | 1 (10) | 6 (27) | 0.123 |
|  |  | Indeterminate | 3 (8) | 2 (20) | 1 (5) |  |
|  |  | Atypical | 15 (42) | 5 (50) | 3 (14) |  |
|  |  | Negative | 13 (36) | 2 (20) | 12 (55) |  |
| May 2020 | n | | 32 | 12 | 15 | - |
|  | Age | | 62 *±* 19 | 56 *±* 18 | 64 *±* 19 | 0.512 |
|  | Male (%) | | 22 (69) | 9 (75) | 6 (40) | 0.101 |
|  | Charlson Comorbidity score | | 2 [1, 6] | 2 [0, 5] | 3 [1, 6] | 0.863 |
|  | Modified Miller score | | 6 [3, 12] | 5 [1, 16] | 7 [2, 11] | 0.871 |
|  | RV/LV ratio | | 0.97  [0.82, 1.25] | 0.99  [0.83, 1.21] | 0.97  [0.84, 1.19] | 0.987 |
|  | Right heart strain * | | 9 (28) | 4 (33) | 4 (27) | 0.923 |
|  | CT  COVID-19 | Typical | 6 (19) | 2 (17) | 4 (27) | 0.779 |
|  |  | Indeterminate | 5 (16) | 1 (8) | 1 (7) |  |
|  |  | Atypical | 16 (50) | 5 (42) | 8 (53) |  |
|  |  | Negative | 5 (16) | 4 (33) | 2 (13) |  |

** RV/LV ratio >1.2*

*Number (%); Mean ± standard deviation; Median [Interquartile range]. Bold indicates statistical significance (p<0.05).*
